# Structural and biomechanical responses of osseous healing: a novel murine nonunion model

**DOI:** 10.1007/s10195-013-0269-4

**Published:** 2013-08-30

**Authors:** Aditya Chaubey, Brian Grawe, Jeffrey A. Meganck, Nathaniel Dyment, Jason Inzana, Xi Jiang, Camille Connolley, Hani Awad, David Rowe, Keith Kenter, Steven A. Goldstein, David Butler

**Affiliations:** 1 Biomedical Engineering Program, School of Energy, Environmental and Biological and Medical Engineering, University of Cincinnati, 2901 Woodside Dr, Cincinnati, OH 45221-0048 USA; 2 Department of Orthopaedic Surgery, College of Medicine, University of Cincinnati, Cincinnati, OH USA; 3 Department of Biomedical Engineering, School of Medicine and Dentistry, University of Rochester, Rochester, NY USA; 4 Center of Regenerative Medicine and Skeletal Development, School of Dental Medicine, University of Connecticut Health Center, Farmington, CT USA; 5 Orthopaedic Research Laboratories, Department of Orthopaedic Surgery, School of Medicine, University of Michigan, Ann Arbor, MI USA; 6 Present Address: Mazumdar Shaw Center for Translational Research, Bangalore, 560099 Karnataka India

**Keywords:** Bone, Fracture, Nonunion, Critical defect, Healing

## Abstract

**Background:**

Understanding the biological mechanisms of why certain fractures are at risk for delayed healing or nonunion requires translational animal models that take advantage of transgenic and other genetic manipulation technologies. Reliable murine nonunion models can be an important tool to understand the biology of nonunion. In this study, we report the results of a recently established model for creating critical defects that lead to atrophic nonunions based on a unique fracture fixation technique.

**Materials and methods:**

Subcritical (0.6 mm long) and critical (1.6 mm long) defects were created in femurs of 10-week-old double transgenic (Col1/Col2) mice and stabilized using a custom-designed plate and four screws. Four groups were used: normal, sham, subcritical, and critical. Histology (*n* = 3 for each group) was analyzed at 2 and 5 weeks, and micro-computed tomography (μCT) and torsional biomechanics (*n* = 12 for each group) were analyzed at 5 weeks.

**Results:**

Subcritical defects showed healing at 2 weeks and were completely healed by 5 weeks, with biomechanical properties not significantly different from normal controls. However, critical defects showed no healing by histology or μCT. These nonunion fractures also displayed no torsional stiffness or strength in 10 of 12 cases.

**Conclusions:**

Our murine fracture model creates reproducible and reliable nonunions and can serve as an ideal platform for studying molecular pathways to contrast healing versus nonhealing events and for evaluating innovative therapeutic approaches to promote healing of a challenging osseous injury.

**Electronic supplementary material:**

The online version of this article (doi:10.1007/s10195-013-0269-4) contains supplementary material, which is available to authorized users.

## Introduction

Fracture healing is an efficient process that leads to the formation of new bone. However, about 10 % of the estimated 5.6 million fractures that occur in the USA annually lead to nonunions [1, 2]. Expenses related to fractures and associated problems are predicted to increase to over US $74 billion by 2015 [3]. Thus, the socioeconomic impact of these injuries cannot be underestimated.

Previous studies have established rodent models for investigating delayed osseous healing [4–6]. However, these models can be technically demanding [6] and not reproducibly manifest clinically relevant scenarios. Murine models have become particularly attractive with the advent of transgenic animals and genome manipulation techniques, and the availability of gene-targeted antibodies [7, 8]. Bone healing occurs in response to injury and is orchestrated by a series of clearly defined events. Although we do understand the basic mechanisms of bone formation, questions still remain as to why an injury, such as a nonunion, does not display a normal healing cascade.

Our group has been studying spatial and temporal *Col1*/*Col2* expression patterns during healing of soft and hard tissue injuries. We bred a unique double transgenic (DT) mouse, pOBCol3.6GFPtpz (Col1) and pCol2-ECFP (Col2), to study tendon midsubstance and insertion-site injuries [9]. This mouse also offers the potential for tracking molecular events in nonunion bone fractures, as Col1 and Col2 are critically involved in bone healing.

The objective of this study is to determine the efficacy of our model in creating a sustained fracture nonunion that could be utilized in future studies to investigate the biological events governing bone healing. In this study, we utilized a unique plate and screw fixation developed by Goldstein and colleagues at the University of Michigan.

## Materials and methods

### Experimental design

Histology and biomechanics were examined in 36 10-week-old male DT mice (72 limbs), containing the transgenes pOBCol3.6GFPtpz and pCol2-ECFP. Four treatment groups were tested in this study: (1) nonoperated control (normal), (2) surgical sham consisting of internal fixation without fracture (sham), (3) subcritical fracture (0.6 mm long) consisting of a surgically induced transverse fracture with internal fixation (subcritical), and (4) critical fracture (1.6 mm long; exceeding the local bone diameter) with internal fixation (critical). Bilateral surgeries were performed, and the groups were randomized among limbs. Histology of the defects (*n* = 3 for each group) was analyzed at 2 and 5 weeks postsurgery, and biomechanics (*n* = 12 for each group) at 5 weeks postsurgery.

### Surgical procedure

All animal protocols were approved by the University of Cincinnati Institutional Animal Care and Use Committee.

**Fig. 1:**
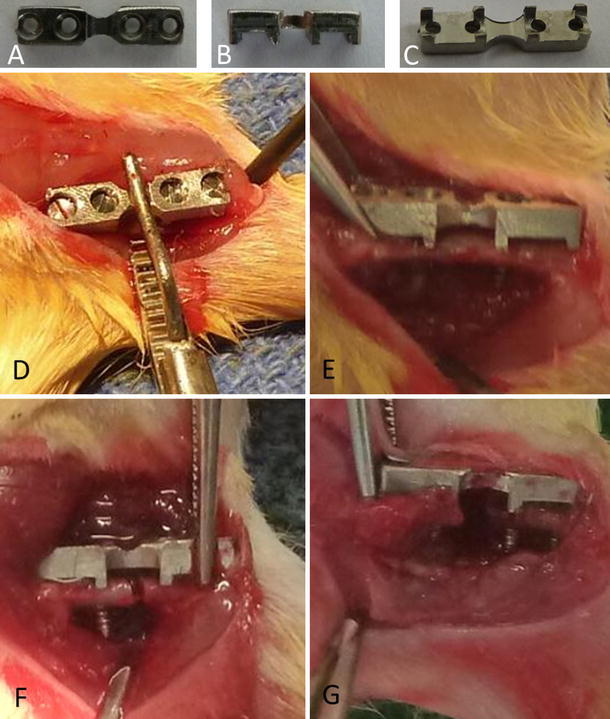
Fracture fixation technique. **a**–**c** Top, side, and bottom views of centrally tapered fracture plate. **d**, **e** The plate is first clamped to the femur, screw holes are drilled, and screws are inserted. **f**, **g** Subcritical and critical defects, respectively

Each animal was anesthetized and maintained on isoflurane. The hair over both hindlimbs was clipped, and they were prepped using chlorhexidine and alcohol. Limbs designated for the normal group were not manipulated. For sham surgery, longitudinal incision was made over the anterolateral aspect of the thigh. The surgeon then applied a novel, centrally tapered four-hole plate to the femur (Fig. 1d, e). Each plate was aligned and centered along the length of the femur using a custom-designed positioning clamp (Fig. 1a–c). The plates allowed for bicortical fixation of four screws, and provided a central taper to allow surgeons to perform an osteotomy. The undersurface of each plate includes a series of centering tabs both to provide alignment and to ensure limited contact with the bone surface. A 0.6-mm-long defect was created in limbs designated for subcritical fracture using a drill-bit after stabilization with the fixation plate (Fig. 1d). In limbs receiving critical-sized fracture defects, 1.6-mm-long defects were created in a band-saw-like fashion using a larger drill-bit (Fig. 1e). All debris was irrigated from the fracture site prior to muscle reapposition. The skin was then closed, and mice were allowed to recover from surgery. After recovery, mice were housed in individual cages and allowed unrestricted activity until sacrifice.

### Histological analysis

Femurs were excised, trimmed of skin, and fixed in 10 % neutral buffered formalin (HT501320.9; Sigma) at 4 °C before shipment to the Rowe laboratory for histological imaging. The fixator device was removed after the femur was radiographed at 24 kV for 8 s with a digital X-ray (LX 60; Faxitron, USA). Subsequently, each femur was soaked overnight in 30 % sucrose/phosphate-buffered saline (PBS) solution, positioned in Neg-50 frozen-section medium (Richard-Allan Scientific, MI), frozen over chilled methylbutane, and kept at −20 °C. Longitudinal full-length 5-μm cryosections were taken either in line with or in cross-section to the screw holes (CM3050S cryostat; Leica, Germany) using a steel blade (cat# 3051835; Fisher Scientific, MA) and nonautofluorescent adhesive film (Section Lab, Co., Ltd., Toyota-gun, Hiroshima, Japan). Three sections were taken at three different depths, and each section was transferred to a glass slide, soaked for 10 min in PBS, stained in 30 mg/mL calcein blue solution (#M1255-1G; Sigma) for 30 min, and cover-slipped with 50 % glycerin in PBS prior to microscopy for the endogenous fluorescent signals.

After imaging of the endogenous signals, the cover-slip was removed and the slide was processed for additional stains. Osteoclasts were identified using fluorescent ELF-97 phosphatase substrate (E6589; Invitrogen), to detect tartrate-resistant acid phosphatase (TRAP) activity. Next, the slide was stained for alkaline phosphatase (AP) activity. After washing in PBS, the slide was re-cover-slipped for imaging using 50 % glycerol containing 10 μg/mL Hoechst 33342 (#H-3570; Molecular Probes). Hematoxylin only or hematoxylin and eosin (H&E) staining was performed on the same slides once the fluorescent staining and imaging were completed. After the cover-slip was removed, the slides were first stained in Myers modified hematoxylin solution (#S216-16 oz; Poly Scientific R&D Corp) for 1 min and then washed with tap water. Then, slides were soaked in bluing solution (#6769001; Shandon) for 2 min and washed with tap water. Slides were then cover-slipped again for imaging.

Fluorescent expression within the femoral sections was examined at 2 and 5 weeks using a Zeiss Mirax Midi scanning fluorescent microscope (Carl Zeiss, Thornwood, NY) and imaged with a high-resolution monochrome digital camera (Zeiss AxioCamHRm). A differential interference contrast (DIC) image was acquired at the same time as the endogenous fluorescence imaging. After detecting bone mineralization with three filters [4′,6-diamidino-2-phenylindole (DAPI), Chroma, #49000ET; Col3.6 blue with cyan fluorescent protein (CFP) (blue), #49001ET; Col3.6 green with yellow fluorescent protein (YFP), 49003 ET], each slide was removed and stained with ELF-97 and reimaged with a yellow filter optimized for tetracycline (Custom HQ409sp, 425dcxr, HQ555/30, set lot C-104285; Chroma Technology). Sections were then stained for: (a) AP activity [fast red substrate with tetramethylrhodamine isothiocyanate (TRITC) filter; Chroma 49005 ET], and (b) hematoxylin imaging (Zeiss AxioCamMRc 5 color camera). The Mirax software created an image stack for each filter setting that could be merged and exported.

### Micro-computed tomography (μCT) imaging and biomechanical analysis

At 5 weeks postoperatively, the femora were excised, trimmed of skin and muscle, and frozen at −80 °C before shipment overnight on dry ice to the Awad laboratory for micro-CT imaging and biomechanical testing. On the day of mechanical testing, each femur was thawed and scanned by micro-CT (VivaCT 40; Scanco Medical, Bassersdorf, Switzerland) at 70 kV_p_ and 145 μA with 300 ms integration time.

Specimens were then hydrated for 1 h in PBS. Each femur was potted in poly(methyl methacrylate) (PMMA) bone cement (DePuyOrthopaedics, Inc., IN) in sections of square aluminum tubing in a custom jig to ensure axial alignment and constant gage length. The bone cement was allowed to set for 2 h prior to rehydration in PBS at room temperature for 1–2 h. Plated specimens were centered in the gage length based on the center of the fixation plate. Nonplated specimens were centered in the gage length based on anatomical landmarks (lateral ridge on the proximal half) for consistency. Prior to testing, the titanium plates were cut perpendicularly through the center using a hand Dremel and a stainless steel, diamond coated disc (Part # 011960U0, Brasseler USA Dental Instrumentation, GA). A #11 scalpel blade was inserted in the space between the bone and plate to protect the femur from the Dremel blade during cutting. Specimens were tested in torsion using an EnduraTec TestBench system (200 N-mm torque cell; Bose Corporation, MN) at 1°/s until failure. Testing was stopped if specimens showed no torsional resistance by 30–40° of rotation.

### Statistical analysis

Biomechanical properties from the normal, sham, subcritical, and critical defects were compared using two-way analysis of variance (ANOVA). Tukey’s honestly significant difference (HSD) post hoc comparisons were made in the event of statistical significance (*p* < 0.05). All data were analyzed using SPSS 13.0 (Chicago, IL).

## Results

### Histological analysis

Figures in this section show representative images for each scenario, for each treatment and time point.

Calcein blue staining revealed Col3.6GFP-positive cells within the metaphyseal bone in normal controls (Suppl. Fig. 1b). Modest GFP signal suggested little endosteal activity in these nearly skeletally mature animals. Osteoclasts (stained by ELF-97 for TRAP) were limited to the metaphysis. Col2A1-positive, blue chondrocytes were scattered but restricted to the articular cartilage and growth plate (Suppl. Fig. 1c). Most bone surfaces showed weak AP staining with DAPI-positive osteocytes within the cortical bone and marrow space (Suppl. Fig. 1d).

At 2 weeks, the cortical bone in the sham-operated control group showed a strong periosteal response around the screw holes (Suppl. Fig. 2a, b). New periosteal bone formation as well as GFP+ bone surface cells (Suppl. Fig. 2c, d) could be seen along the bone surface, especially near the proximal screw hole. Remodeling activity had diminished by 5 weeks, indicating the hardware was well tolerated (Suppl. Fig. 3a, b). Bone cells expressing endogenous Col1 (GFP) were evident, especially around the screw holes (Suppl. Fig. 3b, c). AP staining was visible along the bone and in the growth plates (Suppl. Fig. 3d).

**Fig. 2:**
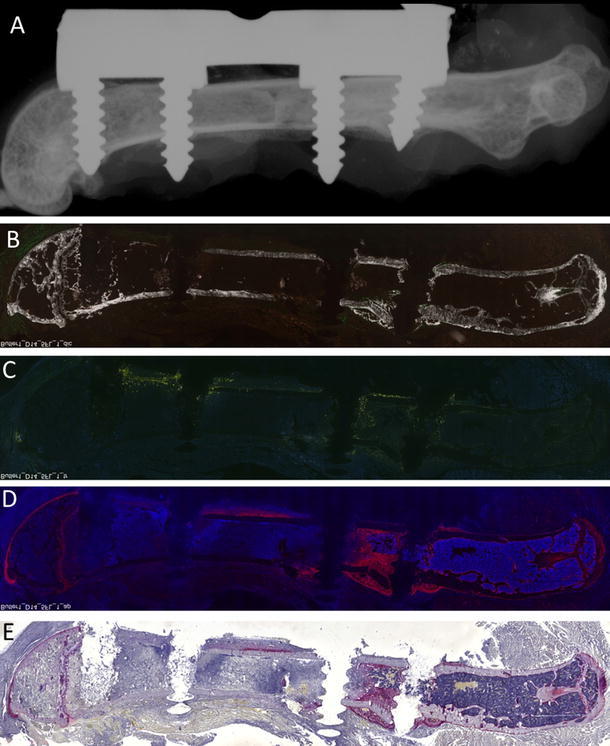
Subcritical defect (0.6 mm) at 2 weeks postsurgery shows adequate healing of the defect. **a** X-ray image of the bone with the plate and the screws intact reveals that bridging has occurred. **b** DIC image showing modest periosteal response. **c** Fluorescent imaging indicates no osteogenic activity on the anterior surface (near to the plate). Positive TRAP staining can be seen in growth plate chondrocytes and cortical bone osteocytes. There is a loss of osteoclastic cells in subendochondral bone, but only a little marrow bone is lost. **d** AP staining (*red*) is evident in the endosteal layer that borders the disrupted bone marrow and the opposite side, but osteocytes are still present in the bone. **e** Hematoxylin staining shows variable quality of the marrow and the presence of debris

**Fig. 3:**
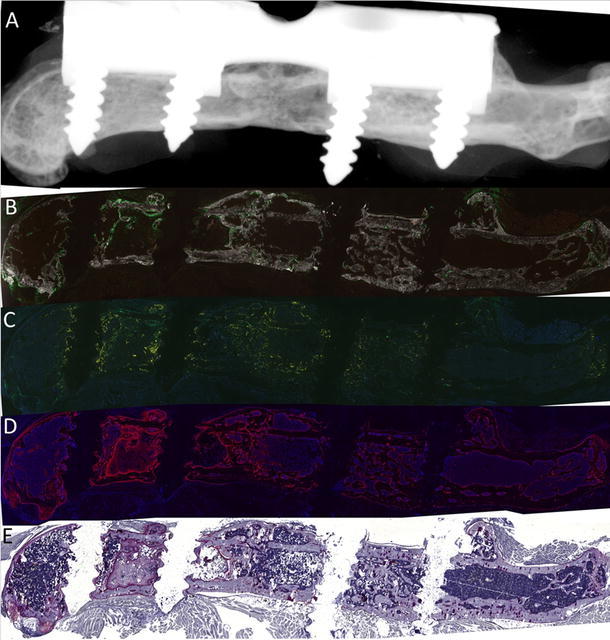
Subcritical defect (0.6 mm) at 5 weeks postsurgery shows adequate healing of the defect. **a** X-ray image of the bone with the plate and the screws intact. **b** DIC image shows that the anterior side (near the plate) is bridged and the posterior side (away from the plate) is spanned by both direct extension and fusion of bone-like trabeculae. **c** Fluorescence imaging reveals that osteoclasts (TRAP+ cells) are attacking original bone beneath the extension/bridging. Bridged section shows some GFP activity. **d** AP staining (*red*) is present in the marrow cavity and especially between the two distal screws (on the *left*). **e** Hematoxylin staining reveals the variable quality of the marrow space

At 2 weeks, in the subcritical defect group, mineral deposition leading to bridging of the bone at the defect site was evident, with only a modest periosteal response (Fig. 2b, c). Although growth plate chondrocytes and cortical bone osteocytes stained positively for TRAP (Fig. 2c), very little osteogenic activity was present, especially on the anterior side (the side away from the plate, Fig. 2c). However, there was evidence of new bone formation around the screw holes (Fig. 2c, d). Subcritical defects had healed by 5 weeks (Fig. 3a). Osteogenic activity (GFP+ cells) was seen at the fracture site (Fig. 3b). New mineral deposition had filled the fracture gap, and islands of disorganized bone had formed in the marrow (defect) space (Fig. 3b). The inner segment (endosteal surface) of original cortical bone was under osteoclastic attack (Fig. 3c). AP staining was present all along the periosteal surface, and especially in the marrow cavity between the distal screws (Fig. 3d). We conclude that subcritical defect healing was evident at 2 weeks and the fracture was complete bridged by 5 weeks.

**Fig. 4:**
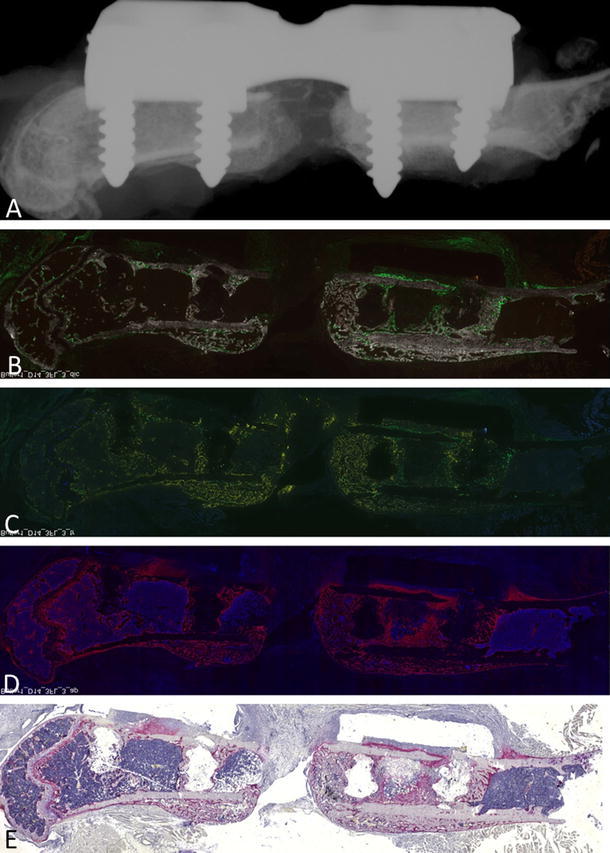
Critical defect (1.6 mm) at 2 weeks postsurgery shows inadequate healing of the defect. **a** X-ray of bone, plate, and screws shows no healing. **b** DIC image also reveals no mineralization. Note the capping of the bone ends on the proximal side. **c** Fluorescence imaging shows capped proximal end, with strong periosteal response on the posterior surface. Positive TRAP staining is seen in growth plate chondrocytes along with some TRAP+ osteocytes. **d** AP staining (*red*) shows active bone around screw holes and along capped end. **e** Hematoxylin staining shows fibrous tissue in defect space with some muscle. Note poor-quality marrow between proximal screws

**Fig. 5:**
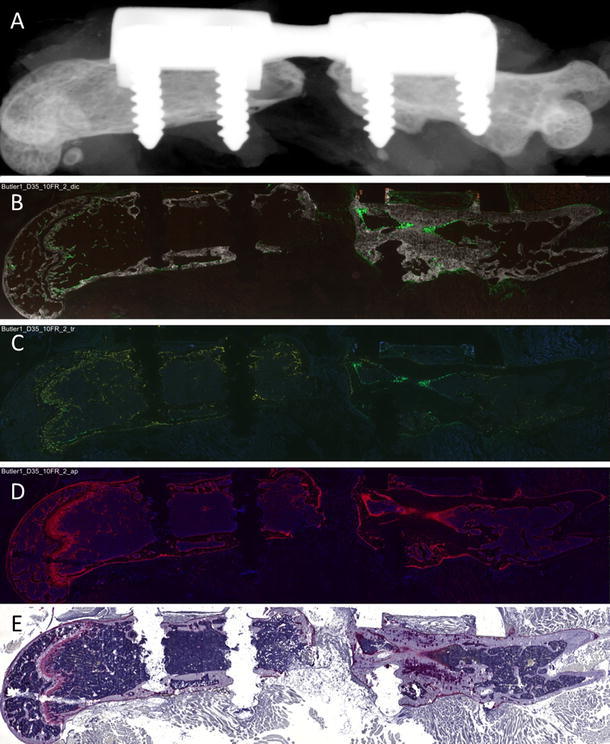
Critical defect (1.6 mm) at 5 weeks postsurgery shows inadequate healing of the defect. **a** X-ray image of the bone with the plate and screws shows no union. **b** DIC image also reveals no mineralization. Capping can be seen on the proximal (*right*) side, whereas the distal side shows partial capping. **c** Fluorescence imaging reveals osteogenic activity (GFP+ cells) around the two proximal screws (on the *right*) and low osteoclastic (TRAP+) activity. **d** AP staining (*red*) is present all along the periosteal surface, in the growth plate and the marrow cavity, and especially between the two proximal screws (on the *right*). **e** Hematoxylin staining reveals healthy marrow space

By contrast, critical defects showed no healing over time. No healing or callus formation was observed at 2 weeks (Fig. 4a). One end of the fracture gap showed virtually no mineralization. The other fracture end was “capped” (Fig. 4b) with osteoclastic activity here and on the periosteal surface away from the plate (Fig. 4c). Osteogenic activity (GFP+ cells) was present along the screw holes (Fig. 4c), and AP activity was detected at the growth plates, all along the periosteal surface, and around the screw holes (Fig. 4d). No healing was evidenced at 5 weeks either. The fractured ends did not show any healing or callus formation (Fig. 5a) due to capping (Fig. 5b). Low osteoclastic activity was detected along the “capped” end, screw holes, and on the periosteal surface of the fracture site opposite the plate (Fig. 5c). Osteogenic activity (GFP+) and AP staining (red) were evident around the screw holes (Fig. 5b, d) with positive AP staining also observed in the growth plate and along the periosteal surface (Fig. 5d).

### Micro-CT and biomechanical analyses

**Fig. 6:**
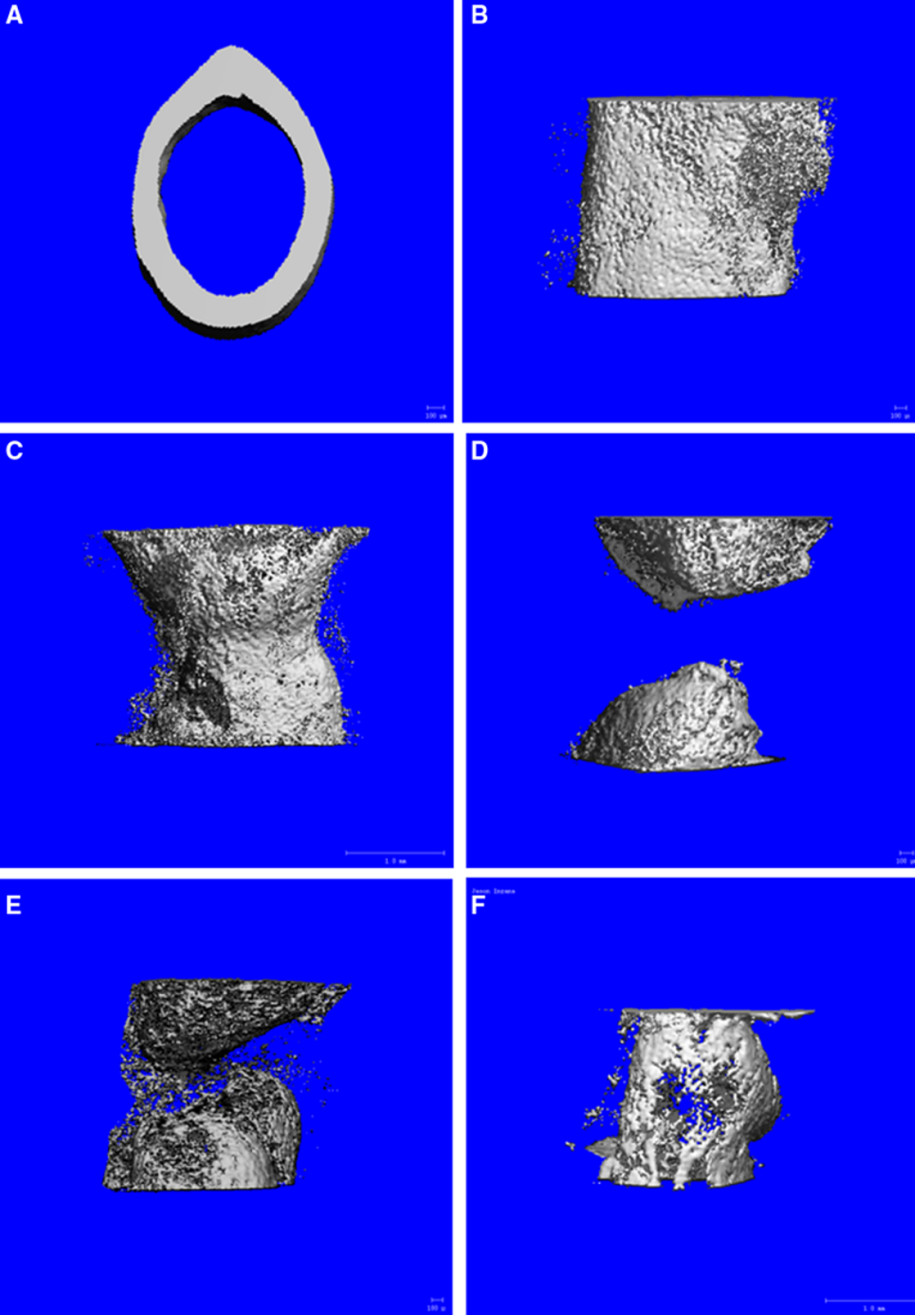
Typical μCT images of **a** normal, **b** sham, **c** subcritical, and **d** critical defects. **e**, **f** μCT images of critical defect samples are consistent with inferior biomechanics

Typical images from volumetric micro-CT imaging are shown in Fig. 6. Qualitatively, we observed dense bone formation in the healing subcritical defects (Fig. 6c), similar to normal (Fig. 6a) and sham specimens (Fig. 6b). By contrast, the majority of the critical defect samples showed either no continuity or healing (e.g., Fig. 6d), or marginal continuity (*n* = 2; Fig. 6e, f).

Biomechanical analysis was performed on 42 of the 48 samples (*n* = 11 each for normal and sham; *n* = 10 each for subcritical and critical). Four samples were lost during mechanical testing (*n* = 2 each for subcritical and critical), and two others were deemed as outliers as their values were more than 2.5 standard deviations (SDs) away from the mean (*n* = 1 each for normal and sham).

**Fig. 7:**
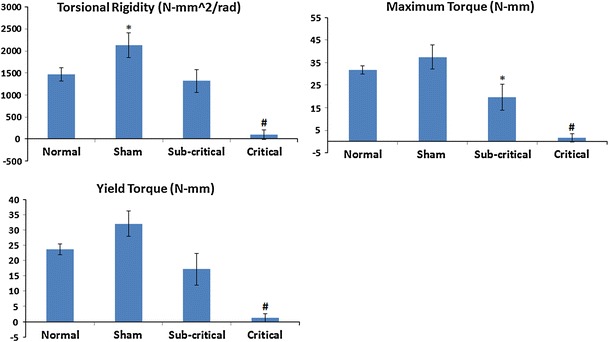
Biomechanical properties (mean ± SD) for subcritical and critical defects.^*,^^#^Values significantly different from normal (*p* < 0.05 and *p* < 0.001, respectively)

Creating critical defects adversely affected mean torsional rigidity (Fig. 7). The torsional rigidity for the critical group samples (99 ± 212.1 N-mm^2^/rad) was significantly lower compared with the results for the normal group (1,464.13 ± 308.1 N-mm^2^/rad; *p* < 0.001; Fig. 7), with only two samples showing measurable torsional resistance. By contrast, the mean torsional rigidity for the subcritical group (1,315.97 ± 525.97 N-mm^2^/rad) was not significantly different from the value for the normal group (*p* > 0.05, Fig. 7). Torsional rigidity for the sham group (2,129.81 ± 564.5 N-mm^2^/rad) was significantly higher than the rigidity for the normal group (*p* < 0.05; Fig. 7).

The yield torque and rotation at yield showed similar patterns to the torsional rigidity results. The yield values (torque, rotation) for the critical group (1.25 ± 2.74 N-mm; 0.002 ± 0.005 rad/mm) were significantly lower than the corresponding values for the normal group (23.7 ± 3.61 N-mm; 0.017 ± 0.002 rad/mm; *p* < 0.001 for yield torque and *p* < 0.05 for rotation at yield; Fig. 7; Table 1). By contrast, yield values for the subcritical group (17.155 ± 10.28 N-mm; 0.016 ± 0.004 rad/mm) were not significantly different from the normal group results (*p* > 0.05, Fig. 7; Table 1). The yield values for the sham group (31.972 ± 8.27 N-mm; 0.020 ± 0.003 rad/mm) were not significantly different from the yield values for the normal group (*p* > 0.05, Fig. 7; Table 1).

**Table 1:** Biomechanical properties (mean ± SD) for subcritical and critical defects

	Rotation at yield (rad/mm)	Rotation at failure (rad/mm)	Energy to failure (N-rad/mm)
Normal	0.017 ± 0.002	0.025 ± 0.003	0.413 ± 0.04
Sham	0.02 ± 0.003	0.027 ± 0.004	0.492 ± 0.17
Subcritical	0.016 ± 0.004	0.021 ± 0.004	0.223 ± 0.14
Critical	0.002 ± 0.005*	0.003 ± 0.01*	0.022 ± 0.07*

* Values significantly different from normal (*p* < 0.05)

Both subcritical and critical defects negatively affected the maximum torque. Values for the critical group (1.6 ± 3.7 N-mm) and subcritical group (19.59 ± 11.44 N-mm) were significantly less than the mean maximum torque for the normal group (31.777 ± 3.47 N-mm) (*p* < 0.001 and *p* < 0.05, respectively; Fig. 7). The maximum torque for the sham group (37.481 ± 10.6 N-mm) was not significantly different from the mean value for the normal group (Fig. 7).

Critical defects also adversely affected failure properties in torsion. The failure values for the critical group were significantly less than the corresponding failure values for the normal group (*p* < 0.05 for rotation at failure and energy to failure, Table 1). Sham and subcritical groups were not significantly different from the mean values for the normal group (*p* > 0.05; Table 1).

## Discussion

In this study, we created a reproducible bone defect healing model to monitor early biological and later biomechanical changes in osseous healing. In our model, subcritical defects were healed by week 5 and were functionally similar to normal controls. By contrast, critical defects did not show any healing by week 5. Histologically, the presence of the “capped” and remodeled fracture ends explains the biomechanical findings. This capping would likely preclude any further boney bridging or osseous remodeling across the fracture site [10]. Biomechanically, critical defects at 5 weeks showed no functional strength. Furthermore, the critical defect group did not demonstrate any progression of healing during serial x-ray examination; in fact, resorption of bone at the ends of the fracture was observed. These findings are consistent with an atrophic nonunion pattern.

The importance of osteo- and chondrogenic cells from periosteum in callus formation following long-bone fractures has been previously demonstrated [11]. Periosteum can drive direct expansion of the original cortical bone without a fibrocartilaginous intermediate stage. We believe this was the mechanism of healing in our model, as we did not see a fibrocartilage core in histology. Endochondral ossification cannot be entirely ruled out, as we did not examine healing earlier than 2 weeks postfracture.

Importantly, periosteal repair is least successful when the fracture ends form a “cap” by migrating inwards. Fibrosis then fills the defect space, and the consequence of this “pseudoarthrosis” is a lack of a periosteal response and new bone formation, the classic “atrophic nonunion.” This phenomenon was seen in critical defects, which displayed “capped” ends.

Our model differs from those of other groups who have reported nonunion models [7, 10, 12]. One advantage of our plate model is the ability to create a reproducible gap that leads to union (0.6 mm) or nonunion (1.6 mm). This can allow for investigation of a variety of treatment strategies ranging from grafts to tissue engineering solutions. It also creates a reproducible volume of repair blastema, enabling molecular and histologic assays. Furthermore, the DT mice offer an ideal model to further study osseous repair, and the response of collagen 1 and 2.

This research is not without limitations. (1) Biomechanical testing was done only at week 5. Since there is no universal definition of nonunions, it has been suggested that they should not be defined arbitrarily in terms of duration, but rather as the ending of the intramembranous healing response [13]. Since the critical defects had “capped” fracture ends, displayed characteristics of true atrophic nonunion, and did not possess any measurable strength even at 5 weeks, we suggest that it is highly unlikely that the critical defects would heal at any later time point, thereby becoming established nonunions. (2) It can be argued that the transgenic manipulation of the mice used in this study could have affected the healing properties. While in most cases a reporter does not have an impact on development and healing, this is still an open question. Since these changes are not readily predictable and highly variable, it will take proper controls in the future to understand their impact, if any, on healing.

Unique in this study is the presence of two fracture models: a subcritical defect that achieves “functional” repair, and a critical defect that does not. We hope to understand cellular and molecular mechanisms that permit repair in one case and then formulate approaches to healing critical defects that lead instead to nonunions. Our long-term goal remains to discover clinically relevant alternatives to treat fracture nonunions by completing fundamental and translational preclinical studies that meet biomechanical and biological design criteria.

## Electronic supplementary material

Below is the link to the electronic supplementary material.

Supplementary material (TIFF 1380 kb)

Supplementary material (TIFF 1996 kb)

Supplementary material (TIFF 1436 kb)

Supplementary material (DOCX 15 kb)
