# Supplementary figures and images for: Structural and biomechanical responses of osseous healing: a novel murine nonunion model
Source: J Orthop Traumatol. 2013 Aug 30;14(4):247–57. doi: 10.1007/s10195-013-0269-4 (PMC3828495; doi:10.1007/s10195-013-0269-4)

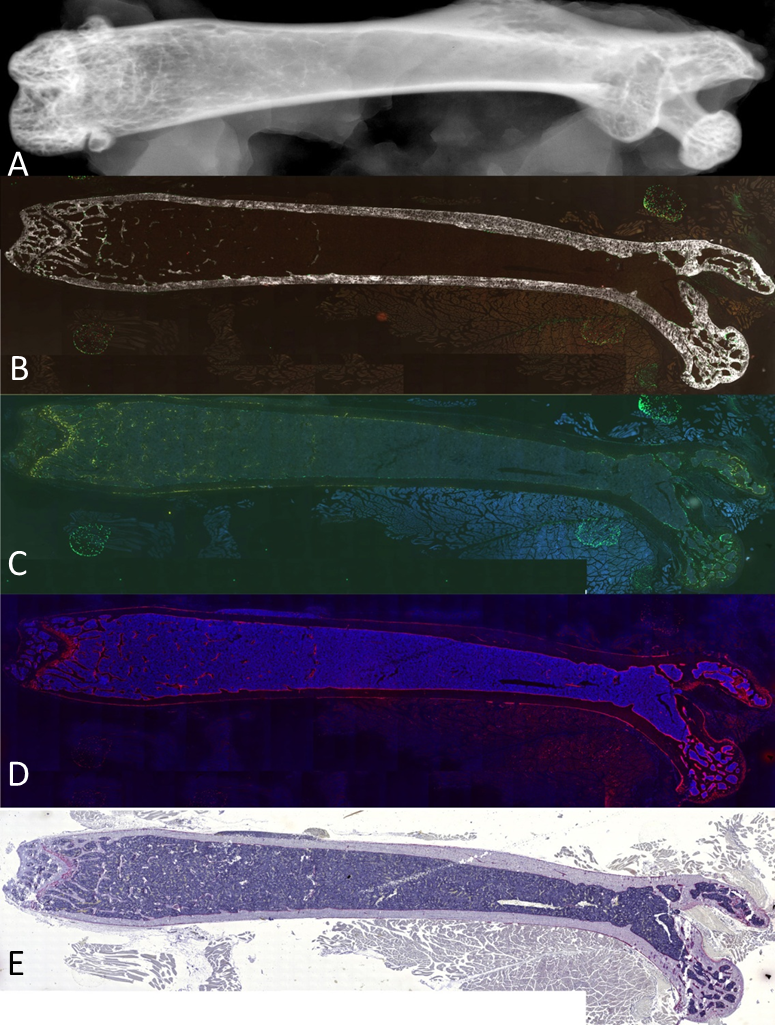

Supplement: Supplementary file 1 — Supplementary material (TIFF 1380 kb) [file 10195_2013_269_MOESM1_ESM.tif]

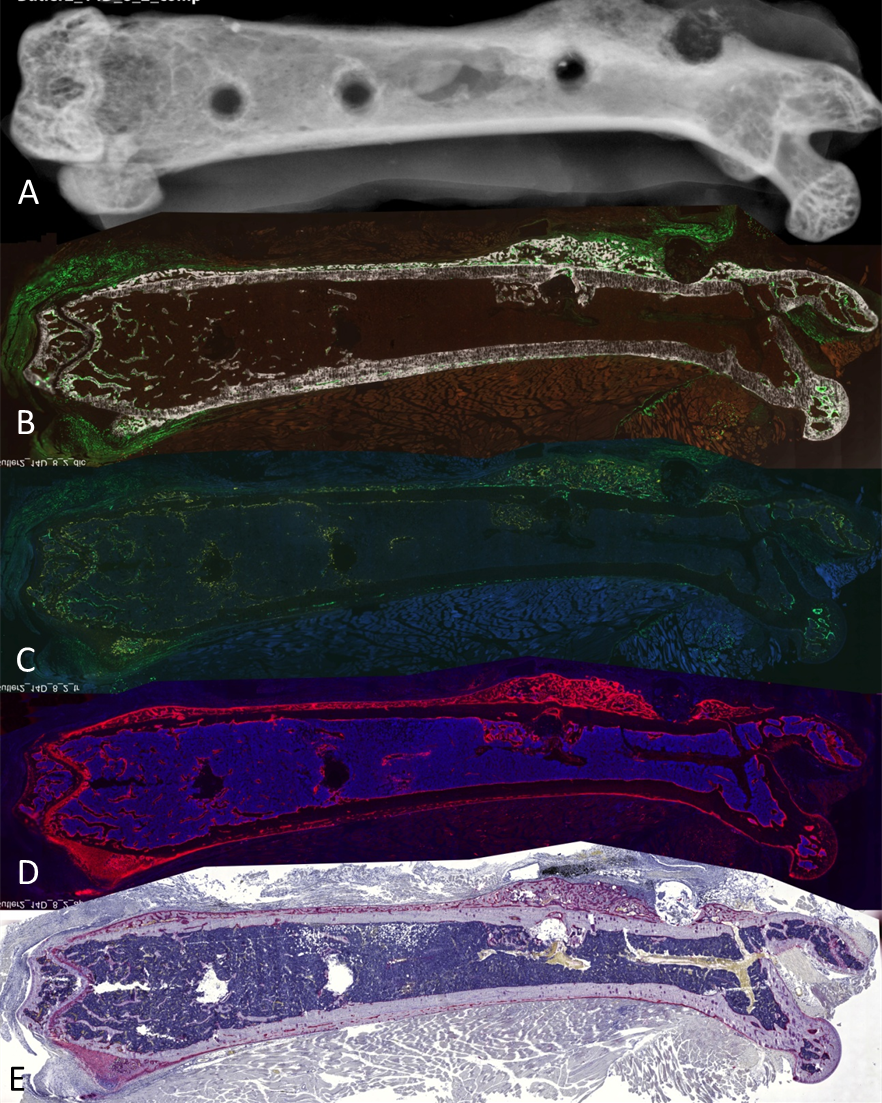

Supplement: Supplementary file 2 — Supplementary material (TIFF 1996 kb) [file 10195_2013_269_MOESM2_ESM.tif]

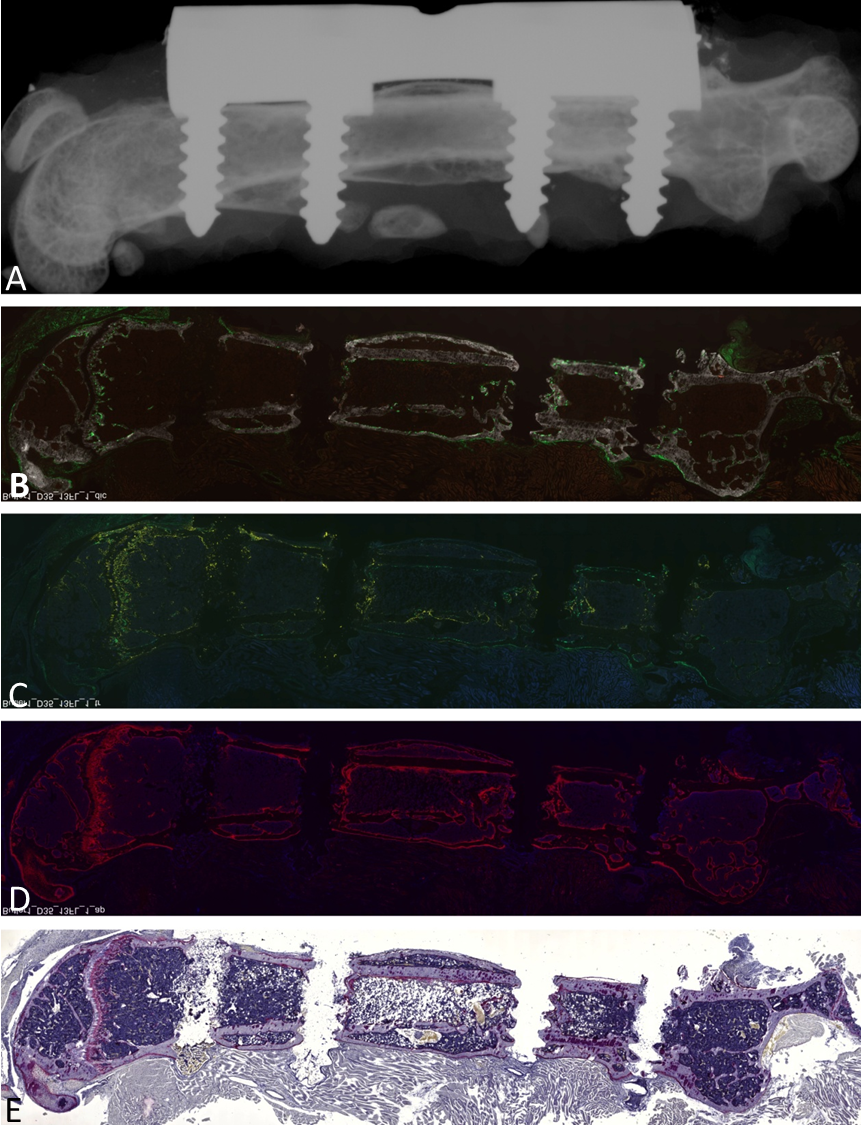

Supplement: Supplementary file 3 — Supplementary material (TIFF 1436 kb) [file 10195_2013_269_MOESM3_ESM.tif]
