# Supplementary material for: Structural and biomechanical responses of osseous healing: a novel murine nonunion model
Source: J Orthop Traumatol. 2013 Aug 30;14(4):247–57. doi: 10.1007/s10195-013-0269-4 (PMC3828495; doi:10.1007/s10195-013-0269-4)
Supplement: Supplementary file 4 — Supplementary material (DOCX 15 kb) [file 10195_2013_269_MOESM4_ESM.docx]

**SUPPLEMENTARY FIGURE LEGENDS**

**Supplementary Figure 1.** Normal femur at 5 weeks shows no osteoblastic activity in the diaphysis. (A) X-ray of femur. (B) DIC imaging reveals mineralized outline in the diaphysis and growth plates. (C) Fluorescence imaging shows endogenous Col1 (GFP) primarily in metaphyseal bone. Some Col2A1 (blue) chondrocytes are seen in the articular cartilage and growth plate. (D) Weak AP staining (red) is seen on most bone surfaces and DAPI-positive osteocytes occur in cortical bone. (E) Hematoxylin staining indicates healthy bone marrow and surrounding skeletal muscle.

**Supplementary Figure 2.** Femurs that were plated, but no defect was created (Sham) at 2 weeks post-surgery. (A) X-ray image of the bone showing that the most proximal screw hole was placed outside the cortical bone. (B) DIC image reveals the presence of new periosteal bone throughout the bone surface with larger outgrowths over the site of the most proximal screw hole. (C) Fluorescent imaging indicates the formation of small amounts of bone around the two distal screw holes. Also visible is significant TRAP accumulation at the site. Note that TRAP positive chondrocytes are present in the growth plate but not in the articular cartilage. (D) AP staining (red) confirms active bone formation around the second proximal screw. (E) Hematoxylin staining shows that the marrow space looks healthy.

**Supplementary Figure 3.** Femurs that were plated, but no defect was created (Sham) at 5 weeks post-surgery. (A) X-ray image of the whole bone with the plate and the screws intact. (B) DIC image shows periosteal new bone formation in response to the plate below the central part of the plate. (C) Fluorescent imaging shows the endogenous Col1 (GFP) and striking TRAP activity in growth plate chondrocytes, indicating damage. (D) AP staining (red) demonstrates bone remodeling around the screws. (E) Hematoxylin staining shows poor marrow in the central section.
